# Orthogonal Experimental Analysis and Mechanism Study on Electrochemical Catalytic Treatment of Carbon Fiber-Reinforced Plastics Assisted by Phosphotungstic Acid

**DOI:** 10.3390/polym12091866

**Published:** 2020-08-19

**Authors:** Chun Pei, Peiheng Guo, Ji-Hua Zhu

**Affiliations:** Guangdong Province Key Laboratory of Durability for Marine Civil Engineering, College of Civil and Transportation Engineering, Shenzhen University, Shenzhen 518060, China; ccpei@szu.edu.cn (C.P.); jason61ban@163.com (P.G.)

**Keywords:** electrochemical catalysis, recycled carbon fibers, orthogonal study, degradation mechanism

## Abstract

Preserving the integrity of carbon fibers when recycling carbon-fiber-reinforced plastics (CFRPs) has been unfeasible due to the harsh reaction conditions required to remove epoxy resin matrixes, which adversely affect the properties of carbon fibers. We establish a practicable and environmentally friendly reclamation strategy for carbon fibers. Carbon fibers are recycled from waste CFRPs by an electrochemical catalytic reaction with the assistance of phosphotungstic acid (PA), which promotes the depolymerization of diglycidyl ether of bisphenol A/ethylenediamine (DGEBA/EDA) epoxy resin. The removal rate, mechanical strength, and microstructure of the recycled carbon fibers are analyzed to explore the mechanism of the electrochemical treatment. The influence of three factors—current density, PA concentration, and reaction time—are studied via an orthogonal method. Range analysis and variance analysis are conducted to investigate the significance of the factors. The optimal conditions are determined accordingly. The underlying CFRP degradation mechanism is also investigated.

## 1. Introduction

Carbon fibers are new high-performance materials with high tensile strength (2–7 GPa), high elastic modulus values (200–700 GPa), low density (1.5–2.0 g/cm^2^), small linear expansion coefficients, and excellent conductivity, and they exhibit good corrosion resistance to acids and alkali and organic solvents [1,2]. To make use of their low weight and high strength, carbon fibers are often combined with polymers to form carbon-fiber-reinforced plastics (CFRPs), which can be used to improve the mechanical and fatigue resistance properties of other materials. Researchers have discovered and developed many excellent properties of CFRPs, such as high toughness, high corrosion resistance, and low weight, and CFRPs are favored by many industries and production fields, including aerospace [3,4,5], automotive [6,7], civil construction [8,9], and sporting goods industries [10,11]. As the application of CFRP materials continues to increase, the amount of carbon fiber waste generated becomes staggering [12,13]. For both environmental protection and sustainable economic development, CFRP waste must be recycled [14,15].

The main purpose of CFRP recycling is to degrade the epoxy resin and obtain high-value carbon fibers. The existing CFRP recovery methods mainly include mechanical recovery [16,17], pyrolysis [18,19], fluidized bed method [20,21], super/subcritical fluid decomposition [22,23], etc. The mechanical recovery has the advantages low cost and simple operation, but this method needs to crush the size of CFRP waste to 5~10 mm, which reduces the reuse value of the recovered fiber [16,17]. Through pyrolysis, the recovered carbon fiber, which accounts for 50–80% of the strength of the virgin fiber, can be recovered, but poisonous gases will be released [18,19]. The strength of carbon fiber recovered by the fluidized bed method is 25–50% of the strength of the virgin fiber, and the length of the fiber is 5–10 mm [20,21]. The strength of carbon fiber recovered by super/subcritical fluid decomposition is 80–85% of the virgin fiber, and the length can reach 10–50 mm. However, toxic organic solvents are needed and energy consumption is high in this method [22,23]. The use of water as a solvent has many advantages, such as a widely available raw material source, low cost, no pollution, and easily handled reaction products [23,24,25]. With the increasing demand for environmental protection, the decomposition of composite materials by the aqueous phase method will be the main trend in the future. Oliveux [26] recycled glass fibers from unsaturated polyester resin under subcritical conditions. Their study revealed that the surface conditions of the recycled glass fibers were well maintained, enabling full reuse. Yuan [27] also successfully recycled glass fibers from a poly(hexahydrotriazine) resin matrix.

In our previous research, a green, energy-saving, and efficient CFRP waste electrocatalytic decomposition recovery method (EHD) based on the degradation mechanism of CFRP was invented [28,29,30]. Sun [29] placed CFRP in 3%, 10%, and 20% NaCl aqueous solutions, respectively, and applied a current of 4–25 mA for carbon fiber recovery with a cycle of 21 d. A small amount of carbon fiber was recovered, and high concentrations of NaCl resulted in severe chlorination and oxidation of the carbon fiber. The tensile strength of the recovered carbon fiber ranged from 32% to 80% of that of the virgin carbon fiber (VCF). Microstructure analysis showed that some epoxy resin residues remained on the carbon fiber surface. The study verified the feasibility of the electrochemical recycling method of carbon fiber under the condition of atmospheric pressure. The utilization of conventional non-toxic chemical solvents and equipment without generating dust, harmful gases or other secondary pollution greatly reduces the recovery cost and initial investment. However, the recovery time needs to be shortened and the tensile strength of the carbon fiber removal rate of epoxy resin needs to be improved. Zhu [30] investigated the effects of varying the applied current density, electrolyte concentration, catalyst concentration, and temperature on the mechanical properties and microstructures of the recycled CFs. With the optimization of parameters, the removal rate of epoxy resin was close to 100% and the average residual tensile strength and interfacial shear strength were approximately 90% and 120% of those of virgin CFs, respectively. This method is a simple, environmentally friendly process with low equipment requirements and can, therefore, reduce the size limit of CFRP waste and be used on a large scale.

Although previous research on water-phase CFRP recovery has led to a series of achievements, it shows that the applied current density strongly influences the quality of recycled carbon fibers [28,29,30,31]. The current density must be precisely controlled to obtain recycled carbon fibers with the same mechanical properties, which requires precise process control. In most cases, the mechanical properties of recycled carbon fibers are normally distributed and can be applied to non-critical parts in the aeronautics, automotive, and architectural industries [32]. Therefore, we want to explore an electrocatalyst that is less sensitive to current density to enable easier operation in practical engineering applications. Besides, the interaction between the factors and the kinetics and mechanism of epoxy resin degradation in the process of recycling needs to be solved.

Heteropoly acids (HPA) are a new class of catalytic materials that have attracted the attention of researchers [33,34]. Heteropoly acids have a high dissociation constant, high ionic conductance, excellent oxophilicity, and good thermodynamic stability. Extensive research on heteropoly acids has led to heteropoly anions with potential applications as electrocatalysts [35,36]. Phosphotungstic heteropoly acid, which is an oxygen-containing acid consisting of phosphorous and tungsten atoms linked by oxygen atoms, is one of the most widely used heteropoly acid catalysts. Phosphotungstic heteropoly acid can react in both the homogeneous phase and the heterogeneous phase and even as a phase-transfer catalyst, thereby making this acid a promising green catalyst for use in aqueous-phase systems [37].

In this study, the electrochemical catalytic method for the depolymerization of epoxy resin and the reclamation of CFRP in phosphotungstic acid (PA) aqueous solution is proposed. Orthogonal test analysis was used to demonstrate the interactions among the reaction parameters reported in previous studies to influence the recovery efficiency: current density, PA concentration, and reaction time. The fundamental degradation mechanism of CFRPs was also investigated via thermogravimetric analysis (TGA), scanning electron microscopy (SEM), atomic force microscopy (AFM), X-ray photoelectron spectrometry (XPS), and gas chromatography–mass spectrometry (GC–MS).

## 2. Materials and Methods

### 2.1. Materials

PA (H_3_[P(W_3_O_10_)_4_]∙12H_2_O), hydrochloric acid (HCl), sodium hydroxide (NaOH) and sodium chloride (NaCl) were of analytical grade and were purchased from Sigma-Aldrich (St. Louis, MO, USA). CFRPs in form of unidirectional carbon fiber pultruded plate were provided by Nanjing Hitech Composites Co., Ltd. (Nanjing, China). The main components of the epoxy resin used in the CFRPs were diglycidyl ether of bisphenol A/ethylenediamine (DGEBA/EDA). The glass-transition temperature of the initial CFRPs was measured by a NETZSCH (Selb, Bavaria, German) STA409PC comprehensive thermal analyzer and reported in Supplementary Information. The information of the initial CFRPs was shown in Table 1.

### 2.2. Test-Specimen Preparation

**Figure 1:**
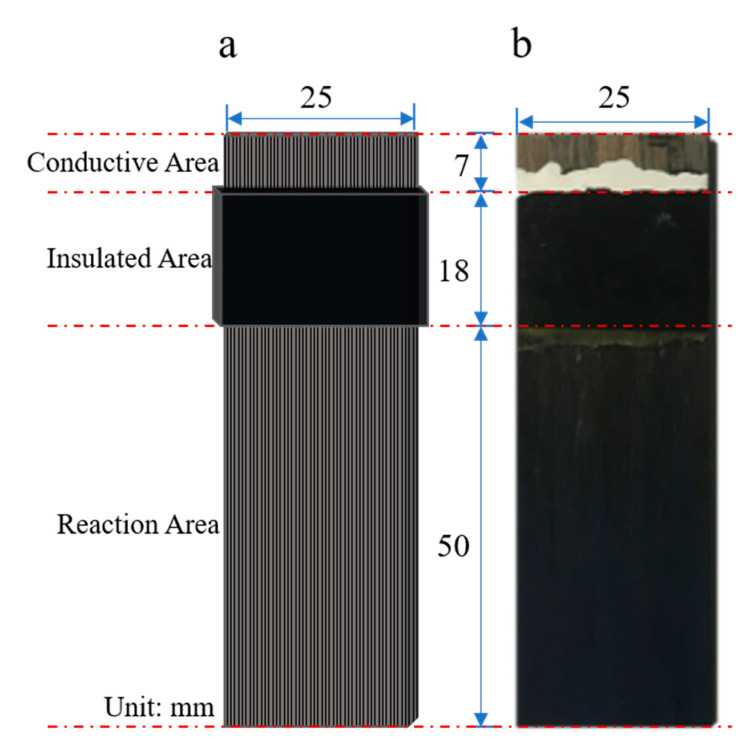
The schematic diagram (**a**) and actual image (**b**) of CFRP test specimen.

The dimensions of each CFRP test specimen were 75 mm × 25 mm × 3 mm, and each specimen was divided into three parts: the reaction area, the insulated area, and the conductive area. A schematic is shown in Figure 1a, and an actual specimen is shown in Figure 1b. First, the surface of the clean and dry CFRP specimen insulation layer was coated with Kraft silicone rubber with a uniform thickness of approximately 1.5 mm, which was then air-dried in the laboratory for 24 h under dry and ventilated conditions. The silicone rubber surface was then wrapped with insulating tape the same width as the insulating layer. The test specimen was then sealed with epoxy sealant and dried for 24 h under dry and ventilated conditions. The main functions of the insulation layer were to control the reaction area of the specimen and isolate the electrolyte from the non-reaction area. The sizes of the reaction area, insulated area, and conductive area were 50 mm × 25 mm, 18 mm × 25 mm, and 7 mm × 25 mm, respectively.

### 2.3. Electrocatalytic Procedures

**Figure 2:**
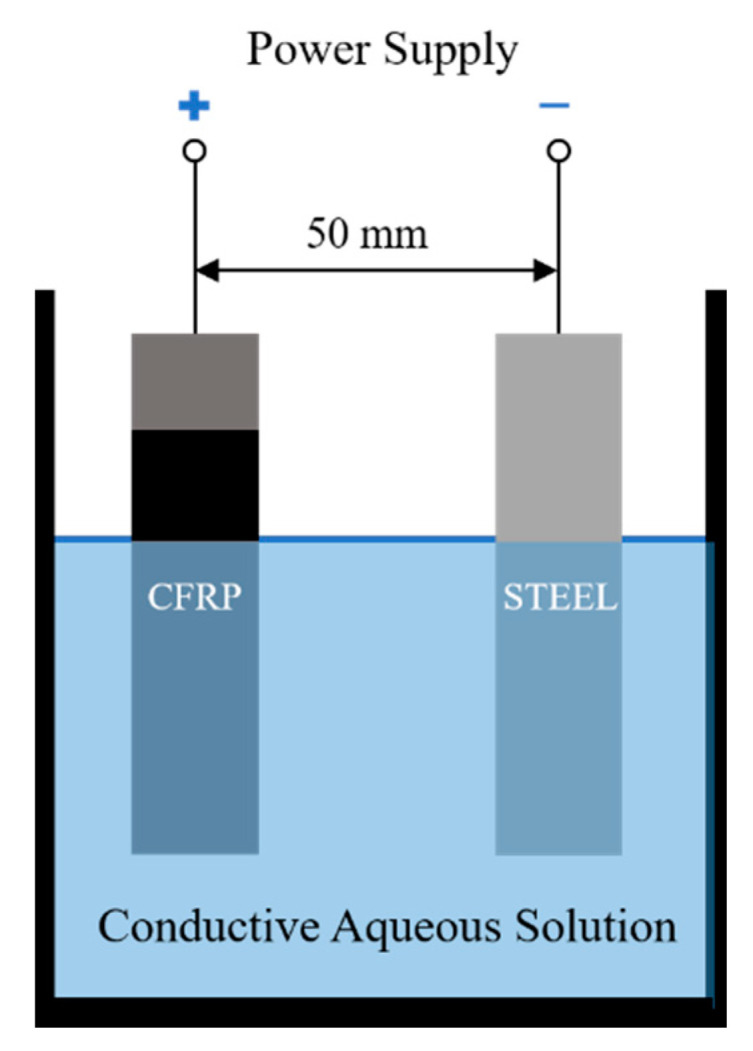
The schematic diagram of the electrochemical catalytic reaction setup.

The setup for the electrochemical catalytic process is shown in Figure 2. The CFRP and stainless-steel plate were connected to an external power supply as the anode and cathode, respectively. Both the CFRP and stainless steel were placed in a container with a conductive solution composed of NaCl, PA, and deionized water. The distance between the cathode and anode was 50 mm, and the reaction temperature was 25 °C. During the electrocatalytic process, the epoxy resin in the CFRP depolymerized and dissolved in the solution; this process enables the recycling of the carbon fibers. HCl and NaOH were used to maintain a consistent pH of 4.5 since PA decomposes at pH values greater than 5.2 [38,39]. The byproducts in the solution may affect the conductivity and consequently change the voltage of the circuit. The electrocatalytic reaction system was controlled at a constant current. A multimeter (MS8261, MASTECH, Dongguan, China) was used to monitor the current and voltage of the system. After the completion of the electrocatalytic reaction, the carbon fibers were cleaned 3 times with deionized water and ethanol in an ultrasonic bath, where each cleaning cycle lasted for 8 min. The carbon fibers were then dried at 60 °C for 24 h to obtain the recovered carbon fiber samples.

### 2.4. Design of Orthogonal Experiment

To investigate the effect of electrocatalytic recycling on the recovery efficiency of CFs, three factors were considered, including current density (A), PA concentration (B), and reaction time (C). Each factor included four levels: A: 3, 4.5, 6, and 7.5 A/m^2^; B: 0, 1, 2, and 3 g/L; C: 24, 48, 72, and 96 h. The L_16_(3^4^) matrix (Table 2) was used to observe and optimize the impacting parameters in the assessment of the effect of the experimental error on the outcomes [40,41]. Each group of experiments was repeated three times.

### 2.5. Characterization Instruments

A STA409PC (NETZSCH, Selb, Bavaria, German) comprehensive thermal analyzer was used for TGA of the recycled carbon fibers under ambient pressure in the temperature range between 30 °C and 800 °C at a controlled heating rate of 10 °C/min. Three samples were tested for each recycling working condition, and the average was reported. Agilent T150 UTM nanomechanical tensile tester with UTM-Bionix Standard Toecomp Quasistatic test system (Palo Alto, Santa Clara, CA, USA) was used to conduct the monofilament tensile tests of the recycled carbon fibers, with a 750 μN loading, 0.2 μm/s tensile rate, 50 nN loading resolution, <0.1 nm displacement resolution, 35 nm tensile resolution, and a ±1 mm maximum displacement of the actuator. The temperature and relative humidity were kept at 20–30 °C and 40% throughout the test, respectively. Twenty replicates were conducted for each recycling working condition. A laser caliper from Changchun Industrial Photoelectric Technology Co., Ltd. (Changchun, China) was used to measure the diameter of the carbon fiber monofilament. The recycled carbon fiber monofilaments were placed on the sample holder. The diameters of the monofilaments were calculated according to the diffraction principle by using the diffraction dark stripe spacing. Twenty replicate measurements were conducted for each recycling working condition. The interfacial shear strength of the carbon fiber monofilament was measured via microdroplet test on an HM410 interfacial evaluation device from Dongrong Industrial Co., Ltd. (Hong Kong, China). The recycled carbon fiber monofilaments were fixed onto the locating hole of concave paper by gluing both ends of the fibers; the paper was then cured for 24 h in a foam plate slot. During the test, the loading speed was 0.12 mm/min and the microscope magnification was 2×. The diameter of the resin drop was measured by a microscope with high magnification before the test. Five filaments were tested for each recycling working condition, and the average was taken for evaluation. The surface morphologies of the carbon fibers were analyzed using an Quanta TM 250 FEG environmental scanning electron microscope (FEI, Hillsboro, OR, USA) with a working distance of approximately 10 mm and an acceleration voltage of 20 kV. An MFP-3d Infinity atomic force microscope from Oxford Instruments (Oxford, United Kingdom) was used to observe the surface morphologies of the carbon fibers. The scanning area was selected to be 1 μm × 1 μm, and the scanning rate was set to 1.0 Hz in tapping mode. Twenty replicate measurements were conducted for each recycling working condition to reduce the experimental deviation. A VPII X-ray photoelectron spectrometer with an aluminum target (ULVAC-PHI, Kanagawa, Japan) was used to obtain the excitation energy peaks of elements. The XPS Peak 4.1 software (Raymund W.M. Kwok, Hong Kong, China) was used to conduct curve fitting of the results. 

The intermediate and final products during the electrocatalytic process were qualitatively analyzed using a 7890a–5975c gas chromatograph-mass spectrometer (Agilent, Palo Alto, Santa Clara, CA, USA). The column was a 30 m × 0.25 mm capillary column with a film thickness of 0.25 µm. The flow rate of the He carrier gas was 1.0 mL/min. The temperatures of the injection port and the transfer line were 250 and 280 °C, respectively. The oven temperature was kept at 70 °C for 1 min and increased to 280 °C at a rate of 10 °C/min. Samples of 2 µL were injected in the pulsed splitless mode (pulse pressure, 25 psi; pulse time, 1 min; purge activation time, 0.9 min). The mass spectrometer was operated in the electron-impact mode at an electron energy of 70 eV. The ion source and quadrupole analyzer were maintained at 230 and 150 °C, respectively. Data were obtained in the selected ion monitoring/scan mode.

## 3. Results and Discussion

### 3.1. Effect of Electrochemical Catalytic Treatment on Depolymerization of the Epoxy Resin

Based on previous studies [38,39], reaction time, current density and PA concentration were the significant parameters affecting the efficiency of the carbon fiber reclamation. The orthogonal analysis was conducted to study the interrelationship of the three major parameters. Sixteen groups of experiments were carried out to investigate the effect of electrochemical catalytic treatment on depolymerization of the epoxy resin, and the degradation rates of epoxy resin within that reaction time, as calculated from TGA by Equation (1), are shown in Table 3.

(1) $$\eta =\frac{\left(M_{v}-M_{r}\right)/M_{v}}{t}$$

where η is the degradation rate within the reaction time, h^−1^; M_v_ is the mass of epoxy resin in virgin carbon fibers, g; M_r_ is the mass of epoxy resin in recycled carbon fibers, g; and t is the reaction time. Both M_v_ and M_r_ can be obtained from the TGA results. The results in Table 3 indicate that the degradation rates of epoxy resin obtained from the L_16_(3^4^) orthogonal array experiments were in the range 0.0102–0.0329 h^−1^. Thus, the optimal electrochemical catalytic treatment conditions for epoxy removal were a current density of 6 A/m^2^, PA concentration of 2 g/L, and reaction time of 24 h. 

Table 4 shows the range analysis for degradation rate of DGEBA/EDA epoxy resin obtained from Equation (2):

(2) $$\bar{K_{\mathrm{ji}}}=\frac{K_{\mathrm{ji}}}{k_{j}}$$

where $\bar{K_{\mathrm{ji}\;}}$ is the average value of each experimental factor; K_ji_ is the sum of the targeting indexes of all levels in each factor; k_j_ is the total levels of the corresponding factor; and i (i = 1, 2, 3, and 4) and j (j = A, B, and C) are the level number and factor, respectively. 

The ranges of each factor calculated from Equation (3) are listed in Table 4,

(3) $$R_{j}=\underset{}{\max}\left(\bar{K_{\mathrm{ji}}}\right)-\underset{}{\min}\left(\bar{K_{\mathrm{ji}}}\right)$$

where R_j_ is the range of the corresponding factor.

The average degradation rate increased slightly with increasing values of factor A and B but decreased with increasing reaction time, as shown in Table 4. The order of the factor impact was C > B > A according to the R_j_ value of each factor. RC was the maximum among all these ranges, indicating that reaction time was the most significant factor influencing the degradation rates of epoxy resin.

Table 4 also shows the results of analysis of variance obtained using Equations (4) and (5):

(4) $${\mathrm{SD}}_{j}=\frac{1}{4}\overset{4}{\underset{i=1}{\sum }}K_{\mathrm{ji}}^{2}-\frac{{\left(\sum_{i=1}^{16}R_{i}\right)}^{2}}{16}$$

(5) $$F=\frac{{\mathrm{SD}}_{j}}{{\mathrm{SD}}_{e}}\times \frac{{\mathrm{df}}_{e}}{{\mathrm{df}}_{i}}$$

where SD_j_ is the square of deviance of each factor; R_i_ is the degradation rate of epoxy resin of the No. i specimen; SD_e_ is the square deviance of error; df_e_ and df_i_ are the degree of freedom of error and factor, respectively. As for the inspection levels of 0.01, 0.05, and 0.1, the critical values of F_0.01_, F_0.05,_ and F_0.1_ were 9.78, 4.76, and 3.29, respectively. According to the variance analysis, all three factors of current density, PA concentration and reaction time were marked as un-significant. However, among them, reaction time expressed the most significant influence on the degradation rate of epoxy resin. 

**Figure 3:**
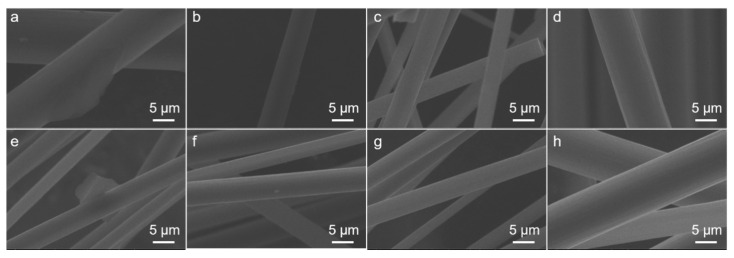
Scanning electron microscopy (SEM) images of the representative recycled carbon fibers. ((**a**) No. 11; (**b**) No. 14; (**c**) No. 3; (**d**) No. 7; (**e**) No. 5; (**f**) No. 2; (**g**) No. 15; and (**h**) No. 12.).

To further investigate the effect of electrochemical catalytic treatment on the degradation rates of epoxy resin, the microstructures of some representative recycled carbon fibers (Nos. 3, 7, 11, and 14 of various reaction times, and Nos. 2, 5, 12, and 15 of various PA concentration) were investigated via SEM analysis and the results are presented in Figure 3. With increasing reaction time, the epoxy resins were gradually degraded and removed from the carbon fiber surface. When the reaction time was 24 h (Figure 3a, representing No. 11), a small amount of resin residue remained on the surface of the recycled carbon fiber. When the reaction time was 48 h (Figure 3b, representing No. 14), the carbon-fiber surface was free from epoxy resin. When the reaction time was 72 h (Figure 3c, representing No. 3), the resin was completely removed. When the reaction time was 96 h (Figure 3d, representing No. 7), no resin remained on the surface of the carbon fiber. However, grooves and cracks appeared on the surface of the carbon fiber, indicating that part of the carbon fiber itself had been electrochemically eroded. 

Figure 3e–h shows SEM images of the recycled carbon fibers obtained from 48 h electrochemical catalytic treatment of various PA concentrations. When no PA was present (PA concentration = 0, No. 5 in Figure 3e), the surface of the recycled carbon fiber was even and smooth. When the PA concentrations were 1 g/L and 2 g/L (No. 2 in Figure 3f and No. 15 in Figure 3g, respectively), no obvious physical defects were observed on the fibers. When the PA concentration was greater than 3 g/L (No. 12 in Figure 3h), strip cracks or concavities on the surface of the carbon fiber gradually began to appear. Thus, the PA concentration of 2 g/L could guarantee the surface quality of the carbon fiber and simultaneously improve the degradation rate. The level of factors not only affected the surface morphology of the recycled carbon fiber but also the mechanical strength, which will be discussed in Section 3.2.

### 3.2. Effect of Electrochemical Catalytic Treatment on Residual Tensile Strength of Recycled Carbon Fibers

The effect of the electrochemical catalytic treatment on the residual tensile strength of recycled carbon fibers was investigated. The tensile strength of the recycled carbon fibers was obtained from monofilament tensile tests and calculated using Equation (6), and the results are shown in Table 3.

(6) $$\sigma_{f}=\frac{F_{f}}{\pi {\frac{D}{4}}^{2}}$$

where σ_f_ represents the tensile strength of carbon fiber monofilament, MPa; F_f_ is the fracture load, N; and D is the diameter of the carbon fiber monofilament, µm, which was obtained by laser caliper. The tensile strengths of the recycled carbon fiber were in the range 861–2688 MPa, whereas that of the virgin carbon fiber was 3277 MPa, which was measured in the same procedure as mentioned in Section 2.5. Thus, the optimal electrochemical catalytic treatment conditions for the residual tensile strength were a current density of 7.5 A/m^2^, PA concentration of 2 g/L, and reaction time of 48 h.

The results of range analysis of the orthogonal tests for the residual tensile strength are listed in Table 5. The order of the factor impact was C > B > A according to the R_j_ value of each factor. R_C_ was the maximum among all these ranges, indicating that reaction time was the most significant factor influencing the tensile strength of the recycled carbon fiber. For the effect of electrochemical catalytic treatment on the residual tensile strength of recycled carbon fiber, a larger index indicates a better result. Since $\bar{K}$_A1_ > $\bar{K}$_A3_ > $\bar{K}$_A2_ > $\bar{K}$_A4_, $\bar{K}$_B3_ > $\bar{K}$_B2_ > $\bar{K}$_B4_ > $\bar{K}$_B1_, $\bar{K}$_C2_ > $\bar{K}$_C1_ > $\bar{K}$_C3_ > $\bar{K}$_C4_, the optimum condition was A_1_B_3_C_2_, which means that the optimum operating parameters for the maximum degradation efficiency were an applied current density of 3 A/m^2^, a PA concentration of 2 g/L and a reaction time of 48 h.

The results of the analysis of variance for the residual tensile strength of recycled carbon fiber were calculated and listed in Table 5. According to the variance analysis, the reaction time was marked as less-significant while the current density and PA concentration were marked as un-significant.

**Figure 4:**
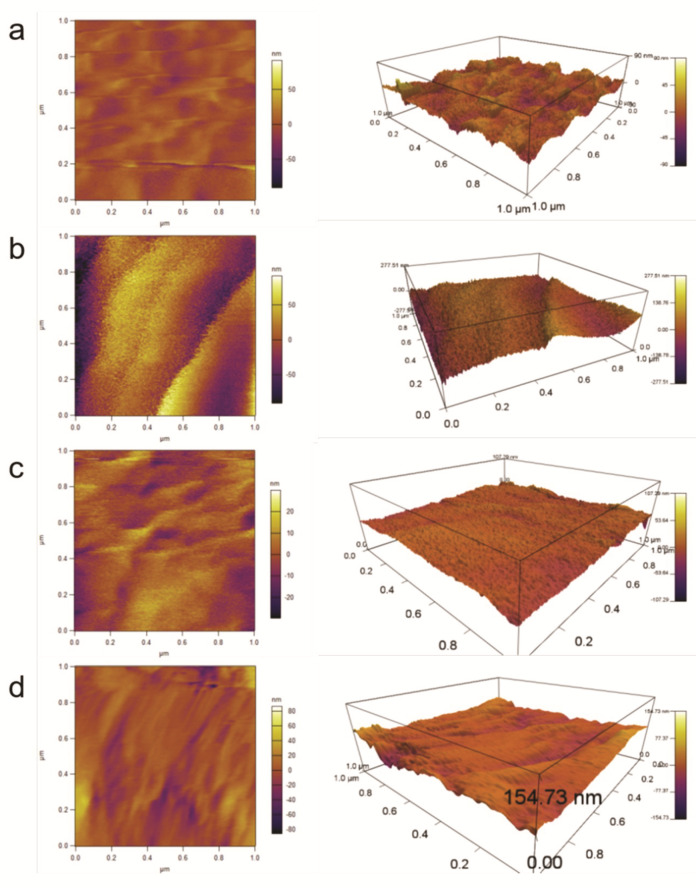
Two-dimensional and three-dimensional atomic force microscopy (AFM) images of the surface morphologies of the representative recovered recycled carbon fibers. ((**a**) No. 5; (**b**) No. 2; (**c**) No. 15; and (**d**) No. 12).

The two-dimensional and three-dimensional surface morphologies of the surface of the recycled carbon fibers at 1 µm × 1 µm under different treatment conditions were obtained from AFM analysis to gain deeper insight into the effect of the electrochemical catalytic treatment on the tensile strength of the recycled carbon fiber. AFM images of sample Nos. 2, 5, 12, and 15 of various PA concentrations treated for 48 h are shown in Figure 4. At low PA concentration (0 and 1 g/L, Figure 4a,b), a small amount of epoxy resin remained after 48 h of reaction, resulting in a rough field surface observed in the AFM images. The cross-sectional area of the measured carbon fiber was larger than the actual cross-sectional area, thus, the calculated tensile strength was relatively lower. With increasing PA concentration, the epoxy resin on the surface of the carbon fiber was gradually removed to reveal a smooth surface of the carbon fiber (Figure 4c). The cross-sectional area measured equaled the actual cross-sectional area of the carbon fiber, and the tensile strength reached a maximum. With a further increase of the PA concentration, the surface of the carbon fiber was scratched and etched by the electrochemical treatment (Figure 4d), which reduced the average cross-sectional area and tensile strength of carbon fiber. The SEM images in Figure 3e–h also show that, when the PA concentration was relatively low (Figure 3e,f), part of the epoxy resin remained on the surface of the carbon fiber. When the PA concentration was 2g/L (Figure 3g), the epoxy resin was completely removed and the carbon fiber was exposed to the electrochemical catalytic reaction environment. When the PA concentration continued to increase (Figure 3f), the surface of the carbon fiber was subjected to electrochemical corrosion, resulting in a decrease in tensile strength.

### 3.3. Effect of Electrochemical Catalytic Treatment on Residual Interfacial Shear Strength of Recycled Carbon Fibers

To investigate the effect of the electrochemical catalytic treatment on residual interfacial shear strength of recycled carbon fiber, the interfacial shear strength of the recycled carbon fiber was tested via microdroplet test and calculated via Equation (7). The results are shown in Table 3.

(7) $$\sigma_{c}=\frac{F_{c}}{\pi \mathrm{dL}}$$

where σ_c_ represents the interfacial shear strength of carbon fiber monofilament, MPa; F_c_ is the fracture load, N; d is the diameter of the carbon fiber monofilament, µm; and L is the diameter of the microdroplet, µm. Notably, the interfacial shear strengths of the recycled carbon fibers were in the range 18–26 MPa, while that of the virgin carbon fiber was 27 MPa, which was measured in the same procedure as mentioned in Section 2.5. Thus, the optimal electrochemical catalytic treatment conditions for the residual tensile strength were a current density of 3 A/m^2^, PA concentration of 2 g/L, and reaction time of 72 h.

The results of the range analysis of orthogonal tests for the interfacial shear strength are listed in Table 6. The order of the factor impact was B > C > A according to the R_j_ value of each factor. RB was the maximum among all these ranges, indicating that PA concentration was the most significant factor influencing the interfacial shear strength of recycled carbon fiber. For the effect of electrochemical catalytic treatment on interfacial shear strength of recycled carbon fiber, a larger index indicates a better result. Since $\bar{K}$_A3_ > $\bar{K}$_A1_ > $\bar{K}$_A4_ > $\bar{K}$_A2_, $\bar{K}$_B3_ > $\bar{K}$_B2_ > $\bar{K}$_B4_ > $\bar{K}$_B1_, $\bar{K}$_C3_ > $\bar{K}$_C2_ > $\bar{K}$_C4_ > $\bar{K}$_C1_, the optimum condition was A_3_B_3_C_3_, which means that the optimum operating parameters for the maximum degradation efficiency were an applied current density of 6 A/m^2^, a PA concentration of 2 g/L and a reaction time of 72 h.

The results of the analysis of variance for the residual tensile strength of recycled carbon fiber were calculated and listed in Table 6. According to the variance analysis, PA concentration, and reaction time were marked as less-significant, while current density was marked as un-significant. Among the three factors, PA concentration expressed the most significant influence on the interfacial shear strength of the recycled carbon fibers.

**Figure 5:**
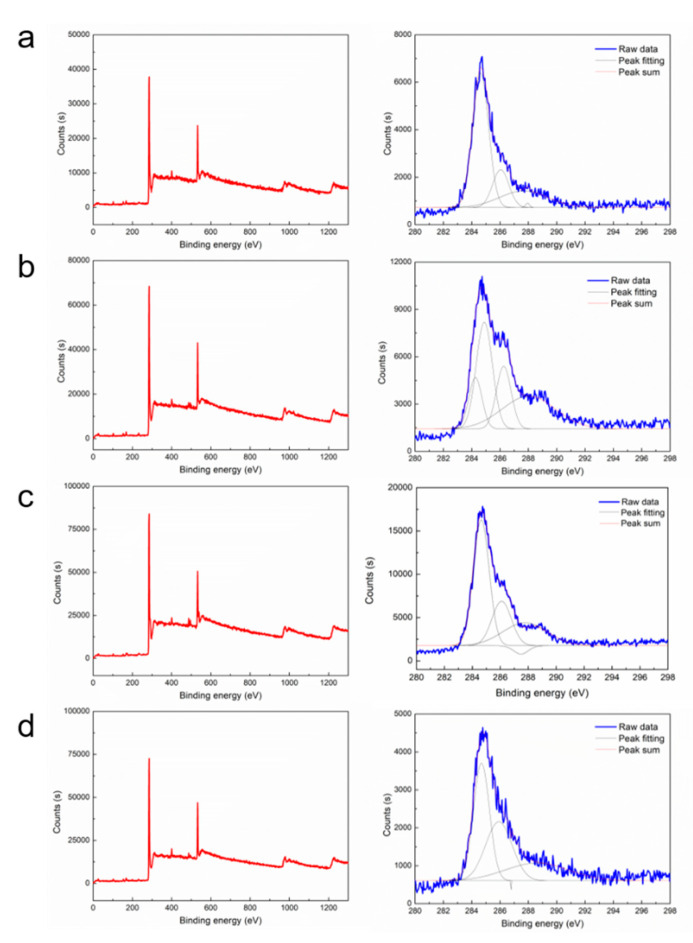
The XPS spectrum of the representative recycled carbon fibers. ((**a**) No. 5; (**b**) No. 2; (**c**) No. 15; and (**d**) No. 12.).

The full spectra and C1s XPS analysis of the carbon fiber recycled under different treatment conditions were collected to further investigate the effect of electrochemical catalytic treatment on the interfacial shear strength of the recycled carbon fibers. The full spectrum and C1s spectrum of samples No. 2, 5, 12, and 15 treated with various PA concentrations for 48 h are shown in Figure 5. The XPS Peak 4.1 software was used to deconvolve the C1s peaks of the carbon fiber samples with various PA concentrations, which were divided into the following chemical bond peaks for Gaussian–Lorentzian fitting [42,43] (as seen in Table 7). Peak 1 represents the graphite peak (C–C) with a binding energy of 284.6 eV; peak 2 represents C–OH with a binding energy of 286.0 eV; peak 3 is C=O with a binding energy of 287.5 eV; and peak 4 is C=O representing –COOH and –COOR with a binding energy of 288.9 eV [28,29,30]. A small peak is observed near 288 eV in the C1s spectrum, indicating the existence of an O–C=O bond [44,45,46]. With increasing PA concentration, the interfacial shear strength of the carbon fiber samples increased because the strong oxidizing ability of PA in aqueous solution promoted the formation of oxygen-containing functional groups on the surface of the carbon fiber, thus improving the surface activity of the carbon fiber and enhancing the binding capacity between the carbon fiber and the test microdroplets. The abundance of C–O bonds and C=O bonds increased with increasing PA concentration, while the abundance of O–C=O groups first increased and then decreased with increasing PA concentration. The O–C=O combined with the hydroxyl group on the adjacent C atoms to form CO_2_, resulting in a decrease in the content of O–C=O at high PA concentrations, resulting in a slight decrease in the interfacial shear strength [47,48]. Thus, the optimal PA concentration was determined to be 2 g/L.

The primary optimization level combination of each factor was determined according to the average value of each indicator at different levels and is listed in Table 8. Because the optimization conditions of the three aforementioned indexes were not consistent, the optimal process conditions were determined on the basis of the comprehensive consideration of the primary and secondary relationship of factors. For factor A, the influence on the degradation rate ranked third, which was a secondary factor. In this case, it can be either A_1_, A_2_, A_3_, or A_4_. The influence on tensile strength and interfacial shear strength of factor A also ranked third, which was a secondary factor. At this point, A_1_ and A_3_ were selected, respectively. However, when A_3_ was selected, the tensile strength was reduced by 8.89% compared with A_1_, whereas the interfacial shear strength was increased by 10.8%. Moreover, A1 was the same as A_3_ for the degradation rate index, thus, A_3_ was taken as the optimal condition for factor A. The optimal condition for factor B was B_3_. The influence on the degradation rate and tensile strength of factor C ranked first, while the influence on interfacial shear strength of factor C also ranked third; thus, C_1_ and C_2_ were selected. When C_2_ was selected, the tensile strength was reduced by 31.5% compared with C_1_, while the degradation rate was increased by 40.6%; therefore, C_1_ was selected for factor C. The optimal combination was selected to be A_3_B_3_C_1_.

### 3.4. The Degradation Mechanism of DGEBA/EDA of Electrochemical Catalytic Treatment

**Figure 6:**
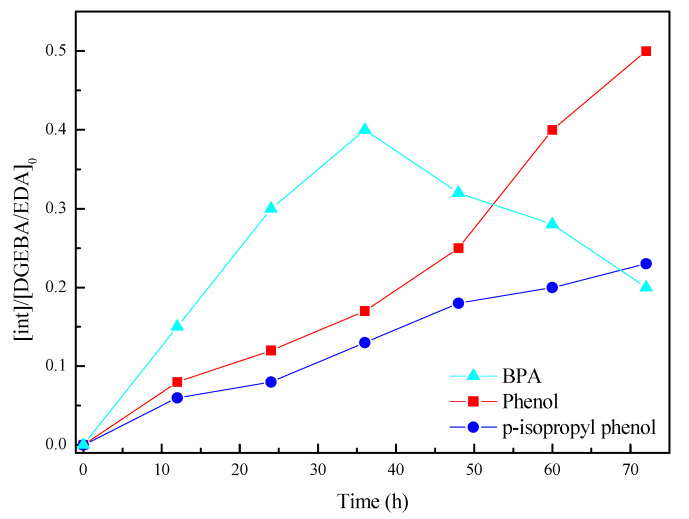
Yields of phenol, p-isopropyl phenol, and bisphenol A (BPA) produced by the degradation of epoxy resin with reaction time.

To conduct a more robust investigation of the depolymerization of DGEBA/EDA epoxy resin during the PA electrochemical catalytic process, the intermediates were identified and the evolution of the major intermediates was investigated. According to GC–MS analysis of the byproducts from the depolymerization of epoxy resin, the intermediate products mainly include phenol, p-isopropyl phenol and bisphenol A (BPA) [49,50,51]. Yield curves of phenol, p-isopropyl phenol and BPA produced by the degradation of epoxy resin concerning the reaction time are presented in Figure 6.

As evident from the curves, the yield of both phenol and p-isopropyl phenol show an increasing trend with increasing reaction time. The yield of BPA first increased with cumulative reaction time, and after the BPA yield reached a maximum value, the yield decreased. As the reaction time accumulated, the generated BPA partially decomposed, resulting in a decrease in yield. The phenol content was relatively high in the early degradation stage of DGEBA/EDA, and the production of p-isopropyl phenol increased in the middle and late stages of degradation. Because of the multielectron structure of the Keggin-type heteropoly compound PA, it tended to oxidize substances throughout the electrochemical process while itself being reduced. The whole process was reversible and easy to regenerate [39,52,53]. The separation and degradation method of the degraded products of phenol, p-isopropyl phenol, and BPA had been widely studied, such as photocatalytic degradation [54,55], Fenton process [56,57], sonochemical degradation [58], and so on. The mechanism of these reactions was advanced oxidation reactions, which has some similarities with the electrocatalytic reaction mechanism of heteropoly acid. Therefore, the electrochemical catalytic reaction of heteropoly acids was also promising for the degradation of these intermediates.

**Figure 7:**
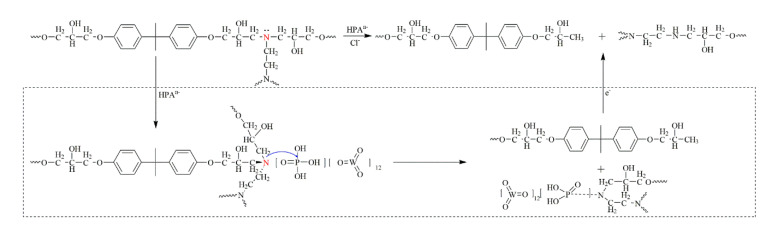
Possible electrochemical catalytic degradation mechanism of diglycidyl ether of bisphenol A/ethylenediamine (DGEBA/EDA) in the presence of heteropoly acids (HPA).

The decomposition of DGEBA/EDA by the electrochemical catalysis of PA might occur through the following stages: HPA disassociated in water to form HPA^n−^ ion. Due to the presence of lone electron pairs on nitrogen, which exhibited a strong electron absorption induction effect, C–N protonation interacted with HPA^n−^ ions to form complexes. The weakly coordinating HPA^n−^ ions coordinated the isolated electron pair of the nitrogen atoms. During the electrochemical process, the now more electrophilic tertiary carbon was attacked, which led to cleavage of the bond between the tertiary carbon and the nitrogen [45,50,59] and resulted in the formation of BPA and ethane diamine (see Figure 7). 

**Figure 8:**
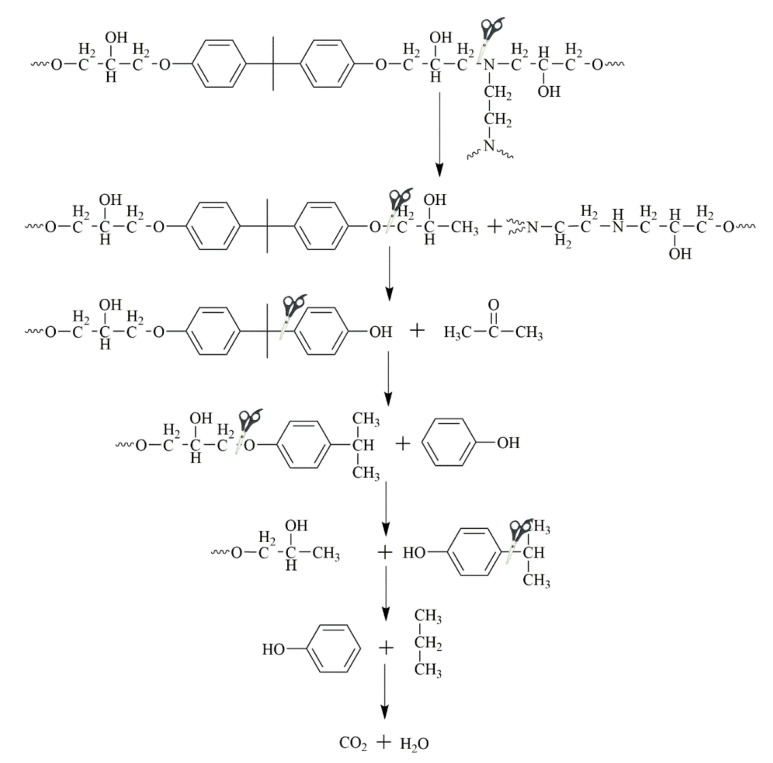
The proposed pathway for the electrochemical catalytic degradation of DGEBA/EDA.

The lone-pair electrons in alkoxy had relatively weak electron-absorbing induction and a strong electron-donating conjugated group when attached to a π-system. However, when such alkoxy groups were attached to saturated carbon, they showed only a simple electron-absorbing effect. The C–O bond could be broken in the same way previously described. The alkoxy para-carbon exhibits electron absorption conjugation and can also form complexes with HPA^n−^. Thus, the C–O bond and C–C bond were broken to form p-isopropyl phenol and phenol [48,60]. The final mineralization of these compounds led to the formation of CO_2_ and H_2_O. The possible pathway for the electrochemical catalytic degradation of DGEBA/EDA is described in Figure 8.

## 4. Conclusions

In this work, we successfully realized the recovery of carbon fiber from CFRP assisted by PA. The effect of an electrochemical catalytic treatment on the degradation rate of epoxy resin and the residual tensile strength and interfacial shear strength of recycled carbon fiber were investigated systematically. Orthogonal experiments were utilized to study the significance and mechanism of influence of the applied current density, PA concentration, and reaction time. The results indicated that, in the electrochemical catalytic process, the epoxy resin was degraded gradually and carbon fiber was subsequently recovered. PA was a promising electrocatalyst for CFRP electrochemical catalytic recovery, and its significance on interfacial shear strength was 2.11 and 6-times greater than that of reaction time and current density, respectively, among the experimental conditions. The effect of reaction time on degradation rate and residual shear strength was greater than that of PA. The degradation rate per unit time decreased with increasing reaction time, and the residual shear strength increased and then decreased with increasing reaction time. Compared with the PA concentration and reaction time, current density was a secondary influencing factor, indicating that the recovered quality was not sensitive to current density, thus, it was easy to manage in practical applications.

## Figures and Tables

**Table 1:** Information of the initial carbon-fiber-reinforced plastics (CFRPs) and CFs.

**CFRP** **Plate**	**Thickness (mm)**	**Width (mm)**	**Length (cm)**	**Tensile Strength (MPa)**	**Tensile Modulus (GPa)**	**Elongation (%)**	**Glass-Transition Temperature (°C)**
	3.0	50	15	≥2800	≥160	≥1.6	135
**CF**	**Mean Diameter (µm)**	**Tensile Strength (MPa)**	**Tensile Modulus (GPa)**	**Elongation (%)**	**Line** **ar** **Density (g/km)**	**Density (g/cm^3^)**	**Weight Content in CFRP (%)**
	7.0	4500	230	1.8	800	1.79	80

**Table 2:** The L_16_(3^4^) orthogonal array for the electrochemical catalytic treatment experiments.

No.	Current Density A (A/m^2^)	PA Concentration B (g/L)	Reaction Time C (h)	No.	Current Density A (A/m^2^)	PA Concentration B (g/L)	Reaction Time C (h)
1	3	0	24	9	6	0	72
2	3	1	48	10	6	1	96
3	3	2	72	11	6	2	24
4	3	3	96	12	6	3	48
5	4.5	0	48	13	7.5	0	96
6	4.5	1	24	14	7.5	1	72
7	4.5	2	96	15	7.5	2	48
8	4.5	3	72	16	7.5	3	24

**Table 3:** Degradation rate of epoxy resin, tensile strength, and interfacial shear strength.

No.	Degradation Rate of Epoxy Resin (h^−1^)	CoV ^#^	Tensile Strength (MPa)	CoV	Interfacial Shear Strength (MPa)	CoV
1	0.031	0.015	1632	0.031	18	0.013
2	0.019	0.016	2615	0.029	25	0.014
3	0.013	0.031	1620	0.028	26	0.022
4	0.010	0.023	910	0.039	22	0.030
5	0.019	0.025	2446	0.026	19	0.044
6	0.032	0.026	1932	0.013	20	0.048
7	0.010	0.029	961	0.029	24	0.036
8	0.014	0.041	1326	0.029	24	0.027
9	0.013	0.026	1302	0.031	21	0.037
10	0.010	0.031	960	0.026	23	0.030
11	0.033	0.003	1922	0.023	26	0.018
12	0.019	0.025	2533	0.027	23	0.028
13	0.010	0.021	861	0.035	20	0.016
14	0.013	0.036	1221	0.031	24	0.019
15	0.019	0.030	2688	0.020	25	0.038
16	0.033	0.026	1554	0.050	20	0.027

^#^ CoV: Coefficient of Variation.

**Table 4:** Range analysis for degradation rate of epoxy resin.

Factors	A	B	C	Error
$\bar{K}$_j1_	0.018	0.018	0.032	0.018
$\bar{K}$_j2_	0.019	0.019	0.019	0.019
$\bar{K}$_j3_	0.019	0.019	0.013	0.018
$\bar{K}$_j4_	0.019	0.019	0.010	0.019
R_j_	0.001	0.001	0.022	0.001
SD_j_	0.000	0.000	0.001	0.30
df_i_	3	3	3	6
F	0.000	0.000	0.007	
	F_0.01_ = 9.780	F_0.05_ = 4.760	F_0.1_ = 3.290	
Significance				

Blank: un-significant.

**Table 5:** Range analysis for tensile strength.

Factors	A	B	C	Error
$\bar{K}$_j1_	1694.5	1560.5	1760.25	1651.75
$\bar{K}$_j2_	1666.25	1682.0	2570.5	1608.0
$\bar{K}$_j3_	1679.25	1797.75	1367.25	1736.50
$\bar{K}$_j4_	1581.0	1580.75	923.0	1624.75
R_j_	113.5	237.25	1647.5	128.5
SD_j_	31002.5	142198.5	5871366.5	39106.5
df_i_	3	3	3	6
F	0.793	3.636	150.138	
	F_0.01_ = 29.500	F_0.05_ = 9.280	F_0.1_ = 5.390	
Significance			*	

*: less significant; blank: un-significant.

**Table 6:** Range analysis for interfacial shear strength.

Factors	A	B	C	Error
$\bar{K}$_j1_	22.750	19.5	21.0	22.5
$\bar{K}$_j2_	21.75	23.0	23.0	22.5
$\bar{K}$_j3_	23.25	25.25	23.75	22.25
$\bar{K}$_j4_	22.25	22.25	22.25	22.75
R_j_	1.5	5.75	2.75	0.5
SD_j_	5	67.5	16.5	0.5
df_i_	3	3	3	6
F	10	135	33	
	F_0.01_ = 29.500	F_0.05_ = 9.280	F_0.1_ = 5.390	
Significance		*	*	

*: less significant; blank: un-significant.

**Table 7:** Contents of surface functional groups of different reaction conditions (Unit: %).

No.	C–C	C–O	C=O	O–C=O
5	65.82	26.60	5.02	2.57
2	66.84	27.22	5.10	2.61
15	59.20	29.55	6.35	4.22
12	56.78	30.12	9.31	3.01

**Table 8:** Primary optimization of process conditions.

Index	Optimal Condition
Degradation rate (h^−1^)	A_(1,2,3,4)_B_(2,3,4)_C_1_
Tensile strength (MPa)	A_1_B_3_C_2_
Interfacial shear strength (MPa)	A_3_B_3_C_3_

## Supplementary Materials

The following are available online at https://www.mdpi.com/2073-4360/12/9/1866/s1, Figure S1. The DSC curve of initial CFRP plate. Table S1. Components of the epoxy resin.
