# Supplementary material for: Orthogonal Experimental Analysis and Mechanism Study on Electrochemical Catalytic Treatment of Carbon Fiber-Reinforced Plastics Assisted by Phosphotungstic Acid
Source: Polymers (Basel). 2020 Aug 19;12(9):1866. doi: 10.3390/polym12091866 (PMC7563943; doi:10.3390/polym12091866)
Supplement: Supplementary file 1 [file polymers-12-01866-s001.pdf]

polymers

Article

Orthogonal Experimental Analysis and Mechanism Study on Electrochemical Catalytic Treatment of Carbon Fiber-Reinforced Plastics Assisted by Phosphotungstic Acid

Chun Pei, Peiheng Guo and Ji-Hua Zhu *

Guangdong Province Key Laboratory of Durability for Marine Civil Engineering, College of Civil and Transportation Engineering, Shenzhen University, Shenzhen 518060, China; ccpei@szu.edu.cn (C.P.); jason61ban@163.com (P.G.)

* Correspondence: zhujh@szu.edu.cn

Received: 02 July 2020; Accepted: 17 August 2020; Published: date

Supplementary information

The glass transition temperature of the initial CFRPs was measured by a NETZSCH (German) STA409PC comprehensive thermal analyzer.

10 mg CFRP samples were weighed each time. The samples were heated from room temperature at a rate of 10°C/min to 200°C, and then kept at 200℃ for 3 min, finally dropped to room temperature at a cooling rate of 40℃/min. The differential scanning calorimetry (DSC) curve was recorded. The glass-transition temperature of CFRP board was determined to be 135℃.

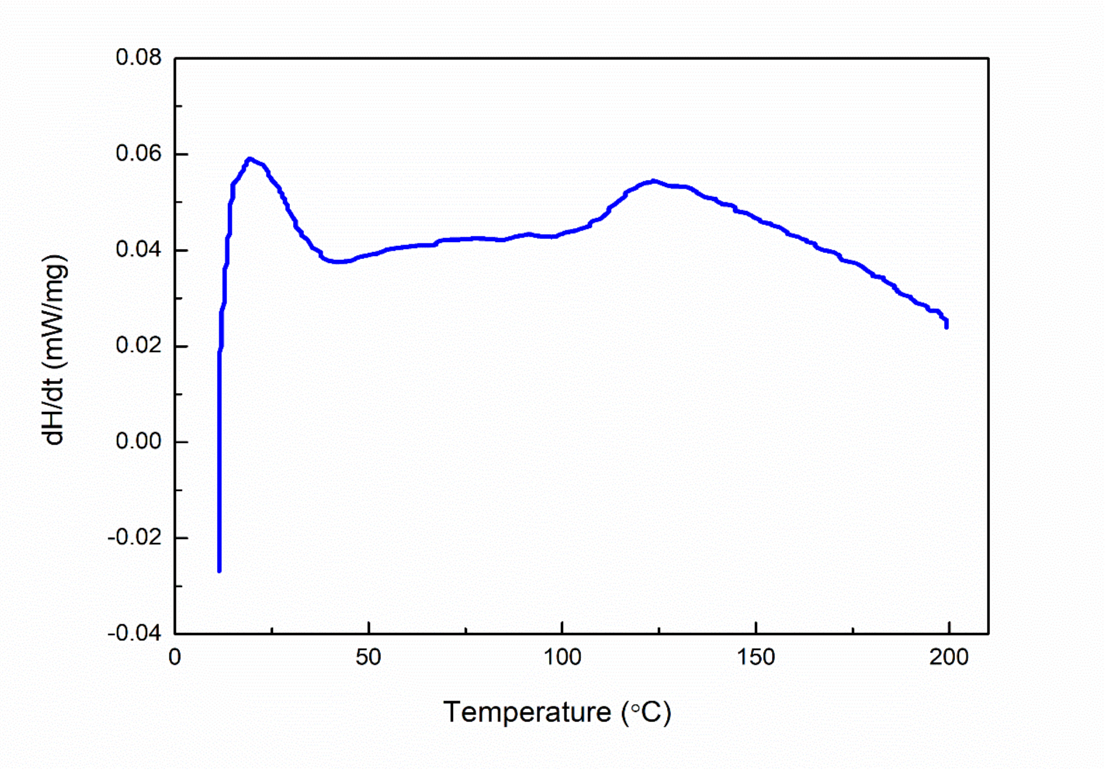

Figure S1. The DSC curve of initial CFRP plate.

The components of the epoxy resin provided by the company are listed in Table SI 1. We are unable to provide the precision data on epoxy resin chemical content. Since the CFRP plates we used are commercial, some of the chemical composition is the trade secret of the company.

Table S1. Components of the epoxy resin.

Components	Mass Fraction (%)
Bisphenol-A-type epoxy resin	67～68
Novolac epoxy resin	9～10
Dicyandiamide	5～6
Methyl ethyl ketone	16～17
